# Economic Stressors, COVID-19 Attitudes, Worry, and Behaviors among U.S. Working Adults: A Mixture Analysis

**DOI:** 10.3390/ijerph18052338

**Published:** 2021-02-27

**Authors:** Andrea Bazzoli, Tahira M. Probst, Hyun Jung Lee

**Affiliations:** Department of Psychology, Washington State University Vancouver, Vancouver, WA 98686, USA; probst@wsu.edu (T.M.P.); hyunjung.lee2@wsu.edu (H.J.L.)

**Keywords:** economic stressors, job insecurity, financial inadequacy, Covid-19, mixture model

## Abstract

Since the unfolding of the novel coronavirus global pandemic, public health research has increasingly suggested that certain groups of individuals may be more exposed to the virus. The aim of this contribution was to investigate whether workers grouped into several latent classes, based on two perceived economic stressors, would report different levels of enactment of the Centers for Disease Control (CDC) recommended behaviors to prevent the spread of such virus. We also tested propositions regarding the potential differential predictors of compliance behavior, differentiating between cognitive (i.e., attitudes toward the CDC guidelines) and affective (i.e., COVID-specific worry) predictors. Using a longitudinal dataset of 419 U.S. workers, we did not find significant differences among the levels of CDC guidelines enactment across three latent classes, representing a range of economic vulnerability. We found that cognitive attitudes were a significantly stronger predictor of compliance with CDC guidelines for workers in the most economically secure class, whereas worry was a significantly stronger predictor of compliance for the most vulnerable counterpart. We discuss these findings in light of the Conservation of Resources theory and other health behavior theories, being mindful of the need to further understand the differential impact of this health and economic crisis on employees facing economic stressors.

## 1. Introduction

The novel coronavirus (2019-nCoV) emerged in late December 2019 and was officially declared a pandemic by the World Health Organization on 11 March 2020. At the time of writing (February 2021), over 99 million people globally have been infected with the disease (COVID-19) caused by the virus and more than two million have died [1]. Despite accounting for only 4% of the world’s population, the United States has accounted for over one-quarter of these cases and deaths. Moreover, research increasingly suggests that certain groups of individuals are more vulnerable to the disease than others, including those who are older and have underlying medical conditions, such as hypertension, diabetes, respiratory issues, and immunocompromised systems. In line with long-standing research on health disparities, communities of color and lower wage workers within the United States have been particularly hard hit by the pandemic as well, suffering higher rates of infections and more morbidity [2].

While understanding prevalence and morbidity rates across different groups is vital to public health, it is also important to understand psychosocial variables that may serve as risk factors for being exposed to or contributing to the transmission of the virus in the first place. Toward that end, the U.S. Centers for Disease Control (CDC) [3] has developed guidance to individuals for reducing person-to-person transmission of the virus, including: frequent handwashing, surface disinfection, staying at home except for essential reasons, maintaining physical separation of at least 6 ft. from others, wearing a cloth facial covering, and covering sneezes and coughs. Yet, emerging data show differences in people’s ability to enact at least some of these behaviors. For example, recent analyses of cell phone location data [4] found that there were higher rates of mobility among individuals living in poorer sections of New York City leading them to conclude that staying home may be a luxury more afforded to individuals living in more well-off neighborhoods. While there are numerous potential reasons for these differences, the data nonetheless suggest that certain groups of individuals may be better equipped than others to follow the CDC guidance.

Therefore, the purpose of the current research was to examine one set of psychosocial risk factors that might predict such behaviors. Specifically, we wanted to examine whether economic vulnerabilities, with respect to job insecurity (fear of job loss) and financial inadequacy (inability to meet one’s financial obligations), are predictive of employees enacting COVID-19 preventative health behaviors, as well as accounting for differences in the extent to which employees worry about COVID-19 and attitudes toward the CDC-recommended preventative health guidelines translate into compliance with those guidelines. To test these propositions, we used data collected during the pandemic to first evaluate hypothesized latent classes of economic vulnerability. Next, we used membership in those latent classes to predict adherence to the CDC-recommended COVID-19 prevention behaviors. Finally, we examined whether individuals belonging to these different classes exhibit different patterns of relationships in their ability to translate worry about COVID-19 and attitudes toward the prevention guidelines into action (i.e., compliance with the recommended behaviors).

In doing so, our study has the potential to provide an important contribution to the literature by: (a) identifying latent classes of economic vulnerability among employees; (b) assessing whether employees in those different classes have different levels of compliance with the CDC guidelines, thereby potentially increasing their risk of exposure to or transmission of the virus; and (c) testing propositions regarding the extent to which employees in those different classes are able to equally translate their worries and attitudes regarding COVID-19 into behaviors that are meant to stem potential transmission of the virus.

## 2. Hypotheses Development

In developing our hypothesis regarding the types of latent classes of economic vulnerability, we drew from the extant literature on economic stressors [5,6], which posits that there are two primary aspects of economic life that serve as potential stressors for employees and their families, namely income-related sources of stress and employment-related sources of stress. Income-related sources of stress include actual economic deprivation (e.g., low income and/or loss of income and financial resources) as well as economic strain (e.g., financial concerns or worries). Employment-related sources of stress include employment instability (e.g., number and duration of periods of unemployment) as well as perceived employment uncertainty (e.g., concerns about possible future layoffs). As can be seen, these stressors can be objective in nature (e.g., household income, periods of unemployment) or subjective in nature (e.g., fears of future job loss or perceived financial inadequacy). Because we were interested in psychosocial risk factors, we primarily focused on the latter subjective stressors in developing hypotheses regarding our latent classes while empirically taking into account more objective aspects, such as income, when testing predicted relationships between membership in those classes and COVID-related attitudes and behaviors.

We used the Conservation of Resources (COR) theory [7,8] as a theoretical foundation for considering how membership in those latest classes may be related to enactment of the CDC-recommended COVID-19 preventative health behaviors. COR theory posits that individuals seek to acquire, protect, and maintain their resources, and will attempt to create circumstances to enable the achievement of these motivations. Such resources can be objects (e.g., house, car), conditions (e.g., stable employment, health), personal characteristics (e.g., self-esteem), or energies (e.g., having available money). Thus, retaining one’s job and having adequate income to meet one’s needs are presumed to be valued resources.

A further proposition of COR theory is that individuals who are lacking in resources will make defensive attempts to conserve their remaining resources. In the context of the coronavirus pandemic, this might lead financially fragile employees to avoid spending additional resources to build an emergency stockpile of groceries, purchasing disinfecting and sanitizing goods, or losing job hours by staying home when feeling unwell, or when a family member is unwell. Similarly, job insecure employees might feel pressured to be present at work despite illness in their household or concerns they may have regarding their workplaces’ ability to provide a safe working environment. On the other hand, employees who feel financially stable and perceive their employment to be secure may be more likely to invest resources toward meeting the CDC-recommended guidelines. Preliminary cross-sectional [9,10] and temporally lagged [11] evidence seems to empirically confirm this proposition, at least at the beginning of the outbreak. Additional longitudinal research has linked precarious work with COVID-related sickness presenteeism [11,12]. On the basis of this theorizing, we expected to find:

**Hypothesis** **1** **(H1).** *Workers belonging to an economically vulnerable class would show lower compliance with CDC guidelines compared to workers in other classes.*

Yet another proposition of COR theory is that individuals with more resources are better positioned for future resource gains (gain spiral), whereas individuals already lacking in resources are more likely to experience future losses (loss spiral). In other words, not only might workers in the most economically vulnerable classes demonstrate less compliance with the CDC guidance, but they might be less able to do so even when they have heightened worries about the COVID-19 virus or have more positive attitudes towards (i.e., desire to) enact the CDC-recommended preventative health behaviors. Thus, they are more likely to exhibit loss spirals in that despite fears regarding the virus or the desire to enact the CDC guidance, they are less able to align their worries and attitudes with their behaviors.

**Figure 1:**
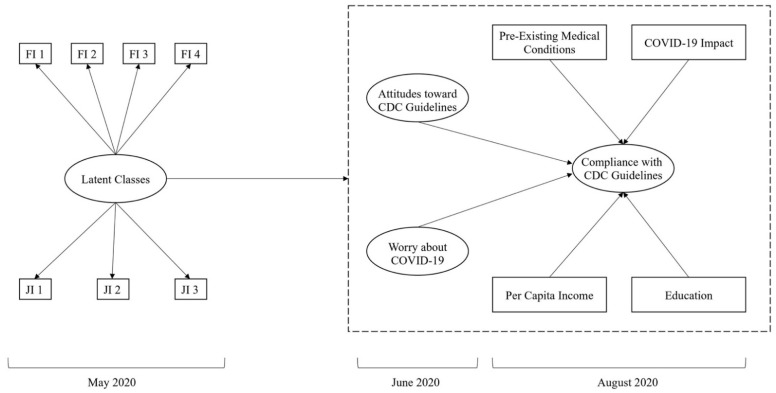
Hypothesized Model. FI = financial inadequacy; JI = job insecurity. The measurement model for attitudes toward Centers for Disease Control (CDC) guidelines, worry of COVID-19, and compliance with CDC guidelines is not depicted for clarity.

On the other hand, workers with secure job and financial stability may be better able to translate their cognitions and emotions into behavior [13]. In other words, employees in the highest resource latent class (i.e., low job insecurity and low financial inadequacy) will be better able to cope with potential stressors (such as COVID-related fears) and translate their attitudes into desired behaviors, whereas the more economically vulnerable classes may not have that luxury. Figure 1 graphically depicts the model that will be tested in this contribution. Thus, we predicted that:

**Hypothesis** **2** **(H2).** *The relationship between attitudes toward the CDC recommended prevention guidelines and compliance with those recommendations will be strongest among workers belonging to the most economically secure class.*

**Hypothesis** **3** **(H3).** *The relationship between COVID-19 worries and compliance with the CDC recommended behaviors will be strongest among workers in the most economically secure class, compared to the other classes.*

## 3. Materials and Methods

### 3.1. Participants and Procedure

A convenience sample of four hundred nineteen U.S.-based adult employees was recruited using Amazon’s MTurk to participate in an ongoing research project entitled “Longitudinal study of work/life experiences during the COVID-19 pandemic.” Employees successively completed three waves of anonymous online surveys in May 2020 (Time 1), June 2020 (Time 2), and August 2020 (Time 3). Participation requirements included (a) being employed outside of MTurk at the time of data collection, (b) having a 90% approval rate or higher for the past 100 crowd-sourced tasks [14], and (c) not having been flagged as a careless respondent in previous waves (i.e., taking on average less than 2 s to answer the survey items, failing two or more attention checks out of four, and being a multivariate outlier; [15]). A breakdown of our sample’s demographics is available in Table 1. There were no significant differences in income (F(2, 418) = 0.12, *p* = 0.89), race (χ^2^(10) = 5.43, *p* = 0.86), or educational attainment (F(2, 418) = 1.75, *p* = 0.18) by gender; however, females in our sample tended to be 4.3 years older than males on average (F(2, 418) = 9.60, *p* < 0.001).

### 3.2. Measures

#### 3.2.1. Time 1: May 2020

Job insecurity was measured using the nine-item Job Security Satisfaction [16]. Participants were asked to indicate on a three-point scale (0: No, 2: Don’t Know, 3: Yes) the extent to which a series of phrases (e.g., “upsetting how little job security I have”) described their current job security. Positively phrased items were reverse-coded so that higher numbers reflected greater felt job insecurity.

Financial inadequacy was measured using Petitta et al.’s four-item scale [17]. Using a 5-point scale (1: never–5: always), we asked the extent to which respondents perceived difficulty in fulfilling their financial obligations. A sample item is “I find it difficult to pay my bills.” The items indicating positive financial situations were reverse-coded such that higher numbers indicated greater financial inadequacy.

#### 3.2.2. Time 2: June 2020

Based on the CDC guidelines to prevent the spread of COVID-19, in place at the time of data collection (CDC, 2020), we developed an 8-item scale asking participants to indicate their attitudes towards social distancing (e.g., maintaining at least 6 ft distance, staying at home) and hygiene practices (e.g., disinfecting practices, hand washing). Respondents answered on a 7-point scale (1: strongly disagree–7: strongly agree). See Appendix A for items.

We measured participants’ worry about catching or spreading COVID-19 and perceived threat to health using three items on a 7-point Likert scale (1: strongly disagree–7: strongly agree). These items were designed keeping in mind the definition of disease-specific worry (McCaul et al., 2020). See Appendix B for items.

#### 3.2.3. Time 3: August 2020

Compliance with CDC guidelines was measured using a six-item measure from [9]. Respondents were asked to indicate how often they were currently engaging in the CDC recommended behaviors on a 7-point scale (1: never–7: always). See Appendix for items.

Control variables. Pre-existing health conditions (e.g., heart disease or condition, high blood pressure, lung/respiratory issues), and the number of governmental COVID-19 policies (e.g., shelter in place, travel restrictions) affecting respondents at the time of data collection were controlled since those are likely to influence individuals’ compliances with the guidelines. Per capita income, measured as the ratio between self-reported total household income bracket (1: less than $10,000–12: $150,000 or more) and number of dependents, and education, measured as the highest degree earned; were also entered in the model.

### 3.3. Data Analysis

Using three job insecurity item parcels and the four financial inadequacy items, we ran several latent classes models. Our aim was to find the adequate number of latent classes with respect to statistical and theoretical criteria. Nylund et al. [18] noted that there is not a fixed criterion to guide class enumeration; rather, a number of statistical tests are available, but the Lo–Mendell–Rubin likelihood ratio test (LMR) [19] seemed to yield consistent results. Additionally, classification accuracy (i.e., entropy) and substantial theoretical value should be taken into account.

In mixture models, indicators are used to identify an underlying latent categorical variable. In the literature, different approaches have been proposed to deal with the issue of continuous auxiliary variables (i.e., other variables, such as covariates and outcomes, which, if included in the latent class estimation procedure concurrently, would lead to an undesirable shift in class estimation; [20]), such as the classify-and-analyze approach, the Lanza method [21], and the three steps approach [22]. The approach developed by Bolck, Croon, and Hagenaars (BCH) [23] has several advantages over other methods proposed [24] and in its simplest implementation the model can be seen as an ANOVA-like model with an observed outcome: this approach estimates the outcome’s mean across latent classes, whose equality is then tested with a Wald’s chi square test. Latent class membership was obtained by weighting the outcome with the inverse of classification error. This model was then used to test Hypothesis 1.

This basic model can be extended in Mplus to include any model [24,25]. Doing so allowed us to test Hypotheses 2 and 3, as these reflect a multigroup model in two steps: first, the latent class model estimation was carried out and the BCH weights were saved, and then, in a second run, the auxiliary model was specified, and the BCH weights used to re-create the latent classes. Both steps were estimated using maximum likelihood with robust standard errors [20].

## 4. Results

### 4.1. Preliminary Analyses

Each of the job insecurity parcels and financial inadequacy items did not approach 3.0 and 10.0 in skewness and kurtosis, respectively, supporting the assumption of univariate normality [26]. We then estimated a confirmatory factor analytical model to test the goodness of our measurement model. Our measurement model (with six correlated residuals) fitted the data well (χ^2^ (308) = 931.08, CFI = 0.90, RMSEA = 0.07, SRMR = 0.07).

Correlations and reliability coefficients are reported in Table 2. As can be seen, job insecurity and financial inadequacy were moderately correlated (*r* = 0.35, *p* < 0.001). Moreover, Time 1 job insecurity was related to less compliance with CDC guidelines at Time 3 (*r* = −0.12, *p* = 0.02). As might be expected, employees reporting more positive attitudes (*r* = 0.69, *p* < 0.001) and greater worry about the pandemic (*r* = 0.56, *p* < 0.001) at Time 2 also reported better compliance with the CDC guidelines at Time 3.

### 4.2. Latent Classes Estimation

Table 3 provides fit indices and estimated class size for the five models that were tested. The benchmarks that were used to evaluate the models included: relative entropy = closest to 1; LMR test = significant (i.e., rejecting the null hypothesis that the *k*-1 model fits better than the *k* model); and substantial theoretical relevance of the subgroups.

Generally, all models showed a comparable, very high entropy. The LMR test showed that the two-class solution fitted the data better than a single-class solution. Additionally, the three-class solution fitted the data better than the two-class model. However, the four- and five-class models did not fit the data better than the three-class solution. Further, the subgroups in the three-class model had substantial theoretical relevance: class 1 represents individual whose economic vulnerability is due mainly to being job insecure, class 2 represents economically secure individuals (i.e., low job insecurity and low financial inadequacy), and class 3 represents the most economically vulnerable individuals (i.e., high job insecurity and high financial inadequacy). For these reasons, we selected the three-class model for all further analyses. Table 4 shows the descriptive statistics from key study variables for participants assigned to a latent class based on their highest posterior probability of class membership.

### 4.3. Tests of Hypotheses

The class-specific mean compliance levels, along with standard errors, are reported in the last row of Table 4. Our first hypothesis predicted that individuals belonging to the least economically vulnerable class (i.e., class 2) would be more compliant with CDC guidelines compared to individuals in the other classes. As can be seen, the most economically secure individuals (i.e., class 2) showed higher levels of compliance behaviors compared to those whose economic vulnerability is due to job insecurity (i.e., class 1; Wald’s χ^2^ (1) = 3.34, *p* = 0.06), but such difference did not reach statistical significance. Similarly, the most economically secure individuals (i.e., class 2) showed higher levels of compliance behaviors compared to the most vulnerable ones (i.e., class 3; Wald’s χ^2^ (1) = 2.39, *p* = 0.12) but the difference was not significant.

Parameter estimates for the structural equation model across latent classes are reported in Table 5. Comporting with Hypothesis 2, Table 4 shows that attitudes were a significantly stronger predictor for class 2 compared to class 3 (*t*_diff_ = 2.70, *p* = 0.007); however, this was not the case for class 1 (*t*_diff_ = 0.81, *p* = 0.42). Further, contrary to Hypothesis 3, results indicate that worry about COVID-19 was a significantly stronger predictor for class 3 compared to class 2 (*t*_diff_ = 2.65, *p* = 0.008), but not compared to class 1 (*t*_diff_ = 1.67, *p* = 0.09). Thus, while attitudes were a stronger predictor of behavior among the financially secure groups, worry was the stronger predictor among the most financially insecure group.

## 5. Discussion

The COVID-19 pandemic has affected human activities worldwide and public health authorities have issued a wide array of regulations and recommendations to slow the spread of the novel coronavirus. However, scholars have noted that economic and public health crises tend to exacerbate pre-existing inequalities [2,27]; hence, already vulnerable populations bear the brunt of such crises. Additionally, the World Bank [28] estimated that between 71 and 100 million people worldwide could be pushed into extreme poverty as a direct result of COVID-19. In fact, organizations both within and outside of the U.S. have called for governments to implement economic measures that would benefit poorer communities [29,30].

Our findings confirmed that our sample could meaningfully be broken down into subgroups according to respondents’ economic vulnerability. In line with the Conservation of Resources theory [7,8], we hypothesized that workers in the most economically secure class would show higher compliance with the CDC-recommended strategies to reduce the spread of the novel coronavirus. However, our results did not lend empirical support to such hypothesis. COR theory posits that the reservoir of resources that is available to individuals and the pathways that are often denied to people with fewer resources is a fundamental determinant of health behaviors. Our results seem—prima facie—to conflict with COR theory; however, unpacking the assumptions of COR theory applied to public health may help in making sense of these results.

The assumption that behavioral change is resource-driven [8,9] is rooted into COR theory; stated otherwise, in order to produce compliance with CDC-recommended guidelines, individuals must be given the resources necessary to implement such behavior. When we collected our data at Time 2 (August 2020), many Americans had received up to $1200 from the federal government under the Coronavirus Aid, Relief, and Economic Security (CARES) Act as means to help them weather the economic crisis generated by COVID-19. By providing money to eligible people, the federal government was in fact providing much needed resources to enable compliance with CDC-recommended guidelines to slow the spread of the novel coronavirus [31], independently of their previous economic vulnerability. Although evaluating whether the amount of money that was disbursed was sufficient to face people’s most pressing issues goes beyond the scope of this paper, and is best left to macroeconomics studies, it will suffice to note that preventative health behaviors do not generally require large sums of money to be implemented, and are far cheaper when compared to other health behaviors [32]. Furthermore, the CARES Act relief check was likely to be more relevant for recipients already in precarious economic conditions compared to those in more financially or job secure situations. In fact, resource gains are known to be taking a greater meaning in the context of resource loss, perhaps acting as a motivator [33]. To further substantiate this claim, data from the Federal Reserve Bank of St. Louis [34] showed that savings increased during the pandemic period, hinting at the possibility that people were using the relief check as emergency money and changing their spending habits to face the economic crisis generated by COVID-19. Hence, contrary to Hypothesis 1, it seems that the workers with the most resources are not necessarily the ones that are the most compliant with CDC guidelines [8,9,31].

### 5.1. Attitudes and Resources

On the other hand, other findings were in line with theoretical expectations based on COR. We found that the more financially secure workers were better able to translate cognitive attitudes into behavior, due to the higher resources available to them, compared to their less financially secure counterparts. Previous research in several fields has found that initiatives targeting cognitive attitudes per se have negligible effects on actual behavior [35]. COR theory purports that the so-called attitude-behavior gap can be bridged only if scholars consider the role of resources that are available to respondents. As Hobfoll and Schumm [8] aptly noted, focusing on resources and environmental circumstances is fundamental when dealing with public health. Not doing so may result in blame-the-victim theories that do more harm than good. Granted that workers with ample resources will be less vulnerable to resource loss spirals [8], our findings confirm the relevance of previous resources in the current health and economic crisis. Scholars cannot sidestep the role of resource reservoir that is available to people and make some courses of action available only to resource-endowed workers, while the same pathways are often denied to those lacking resources that would allow them to pursue such behaviors. Hence, public health programs in response to crises should be focused on (a) providing and mobilizing short-term resources to offset immediate resource loss experienced by communities and workers, and (b) establishing prevention efforts. Arguably, the latter option was historically implemented by European social democracies, which have long relied on extensive social security net protections for workers by providing them with a wide array of tools that will be needed once they become unemployed due to an economic crisis. Preliminary evidence in the U.S. (Probst et al., 2020) seems to suggest a similar pattern: more extensive state-level unemployment benefits weakened the observed negative relationship between job insecurity and enactment of COVID-19 preventative behaviors.

### 5.2. Worry as a Resource

Research in health psychology has long recognized that disease-specific worry motivates preventative health behaviors [36,37]. Among others, worry has been shown to be related to higher job performance [38] and higher problem-solving abilities [39]. Worry’s motivational power is purported to affect behavior via three mechanisms [40]: the most relevant mechanism for the purposes of this contribution posits that worrying about a stressor (e.g., the novel coronavirus) keeps the stressor and its feared outcomes at the forefront of one’s mind, provides frequent and continuous cues to action, and sustains motivation towards action [41]. Our results showed that participants belonging to the most economically vulnerable class were the ones that were motivated the most by COVID-19-specific worry, compared to the other more economically secure classes.

This counterintuitive result may be explained by a combination of pragmatic prospection [42] and COR theory. Worry may be essential to productive planning of the future through thinking about the future in ways that will guide practical behaviors. In our study, the most economically insecure workers were in an already dire situation at the start of the COVID-19 pandemic, and one of their goals, consistently with COR, may be avoiding further resource loss that might occur should they become infected with the coronavirus. Additionally, should they become ill, they would be faced with a host of negative consequences: for instance, current CDC guidelines recommend a 10-day quarantine, which would prevent workers from reporting to one’s job, and would translate into income loss and potential termination. Furthermore, healthcare costs associated with COVID-19 are estimated to be on average over $38,000 for people insured through their employer [43] and over $88,000 for patients requiring respiratory support through a ventilator [44]. This potential financial burden is therefore more likely to be relevant for workers that are already in a precarious economic situation, compared to the more economically secure counterparts, triggering worry toward the novel coronavirus. These findings are in line with extant motivational theories in health psychology [45].

Furthermore, previous quantitative and qualitative research has shown that worry can be meaningfully conceptualized as a flexible resource that may help people in bringing up issues and motivate proactive behaviors to solve such issues [46]. Interestingly, extant research showed that problem-related worry tends to be reported when financial circumstances are not ideal [47] because a subjective need for security is not being met, due to financial hardships, leading to worry. This line of thinking has driven scholars to suggest that worry reported by the most vulnerable makes their decision-making process more shortsighted; thus, creating a feedback loop that perpetuates poverty [48]. Our results, however, seem to suggest that outcome-specific worry can be leveraged as a resource that can motivate behaviors aimed at preventing feared negative consequences, such as catching COVID-19. Since we did not use financial worry as a predictor, but used cognitive perceptions of financial inadequacy, future research should extend our results by considering financial worry as well.

### 5.3. Relevance to Occupational Health and Future Directions

Occupational health research has long investigated the relationship between economic stressors and compliance behaviors, mainly in the realm of safety behaviors [17,49]. Specifically, empirical evidence showed that in an attempt to keep their job and income source, employees may be feeling pressured to forgo safety procedures and prioritize other behaviors (e.g., productivity; [50]) that they perceive their employer values the most. Previous research found that both cognitive (e.g., financial inadequacy [17]) and affective (e.g., affective job insecurity [51]) economic stressors predicted lower safety compliance and more accidents, but the latter are theorized to have a more negative impact [52].

Our findings, however, seem to point to another direction: when it comes to enacting preventive behaviors related to the spread of the novel coronavirus, cognitive attitudes are a relevant motivator only for the most economically secure workers, while the most economically fragile employees seemed to be more motivated to comply with CDC recommendations by worries about catching COVID-19. Although we did not directly measure any safety outcome, we believe that our findings may inform future research on occupational health in the following ways: first, economic stressors seem to influence the motivational processes that drive workers’ behaviors. Insights from this study could be expanded to risk perceptions and motivations to engage in safety behaviors. Specifically, research on the relationship between economic stressors and safety behaviors has focused mainly on so-called job preservation motivations, assuming that the employees’ goal is to keep their job by means of engaging in the behaviors that are valued the most by their employer. Future research should clarify whether employees weigh the benefits of noncompliance with safety norms (e.g., aligning oneself with the perceived organizational priorities and keeping one’s job) more heavily than their health concerns. This decision process may be likely influenced by several other variables germane to occupational health psychology: for instance, research has shown that risk perception is influenced by affect and worry [53]; hence, more economically vulnerable employees may perceive the novel coronavirus as riskier than the more secure counterpart. In turn, this would suggest that they should be more likely to engage in self-protective behaviors (such as safety compliance; [54]) due to the fear of negative health consequences. Other organizational phenomena are also likely to influence the decision-making process [55]: team-level constructs (e.g., safety climate), specific workplace characteristics (e.g., job design), and extra-organizational trends (e.g., industry-wide shifts) may have a role.

### 5.4. Limitations

Although our findings suggest a link between economic vulnerability and the motivations that drive compliance with CDC guidelines, several limitations need to be acknowledged and addressed by future research. First, due to our convenience sampling techniques, we cannot claim to have a representative sample of the U.S. population. Although previous studies indicate that gender and race compositions of MTurk samples approximated the general U.S. population [56,57], they also tended to be younger, more educated, and differed in religious affiliations. Our sample was similarly younger and more educated with a closer approximation, with respect to race and gender (e.g., Whites: 74%, male: 59%) compared to the U.S. workforce (Whites: 78%, male: 53%). Additionally, all participants were employed or furloughed at the time of the first data collection wave.

Another limitation is that we were not able to include any measure of participant workplace conditions. Arguably, future research should investigate whether specific working conditions (e.g., control over implementation of measures aimed at reducing the spread of COVID-19 in the workplace, employer’s ability, and willingness to provide individual protective equipment) have an effect on individual compliance behavior. In fact, the Oregon Occupational Safety and Health Administration received over 1200 complaints in March 2020 alleging that businesses across the state were violating public health measures [58]. Among those, accounts included instances in which employees were forced to work while showing symptoms of COVID-19 and threatened with termination if they called in sick, or were actually terminated for engaging in the CDC-recommended 14-day quarantine after a potential exposure.

Additionally, we were able to include only preventative behaviors in this study. Future research should examine whether differential motivations to engage in CDC-recommended guidelines translates into differential exposure and morbidity. Similarly, we included only relatively short-term behaviors. COR theory suggests that longer-term impacts may be different from those observed in the short-term. For this reason, future investigations of long-term effects of the COVID-19 pandemic are warranted.

## 6. Conclusions

The COVID-19 pandemic has reinforced prior knowledge of economic and health disparities within the United States. The current study adds to this body of literature by examining two pre-existing psychosocial risk factors: job insecurity and financial inadequacy. Specifically, latent class analysis confirms that employees can be meaningfully classified into three groups with varying levels of each economic stressor. Moreover, while membership per se did not predict enactment of the CDC-recommended COVID-19 prevention behaviors, membership was associated with employees’ ability to translate worries about the pandemic and attitudes toward the recommended guidelines into behavior. Both findings highlight the importance of understanding psychosocial factors that may serve as barriers or facilitators of engaging in behaviors meant to stem the tide of the COVID-19 pandemic.

## Figures and Tables

**Table 1:** Sample’s Demographic Characteristics.

Variables	Percentage	n
Gender		
Male	59.5%	249
Female	40%	168
Missing	0.5%	2
Age		
Younger than 25	3%	7
Between 26 and 35	38%	158
Between 36 and 45	32%	132
46 and older	25%	106
Missing	2%	6
Race/Ethnicity		
African American	8%	34
Asian American or Pacific Islander	9%	38
Caucasian	74%	309
Hispanic/Latinx	7%	30
Other Minority	2%	8
Education		
High School Diploma or Less	8%	33
High School Diploma and Technical Training	2%	7
Some College	24%	101
College Degree	48%	200
Some Graduate School	2%	11
Graduate Degree	16%	67
Industry		
Accommodation/Food Services	5%	20
Administration & Support Services	5%	23
Education	9%	40
Finance	9%	39
Healthcare	10%	40
Information	6%	26
Manufacturing	7%	28
Professional, Scientific, or Technical Services	17%	70
Retail	13%	55
Other	19%	78

**Table 2:** Correlations and scale reliability.

	1	2	3	4	5	6	7	8	9
1. T1 Job Insecurity	(0.94)								
2. T1 Financial Inadequacy	0.35 ***	(0.87)							
3. T2 Attitudes	−0.01	−0.09	(0.85)						
4. T2 Worry	0.05	0.11 *	0.62 ***	(0.89)					
5. T3 COVID-19 Impact	−0.11 *	−0.06	0.09	0.15 **	---				
6. T3 Income	−0.09	−0.32 ***	0.08	0.01	0.07	---			
7. T3 Education	−0.07	−0.07	−0.01	0.04	0.20 ***	0.22 ***	---		
8. T3 Health Conditions	0.03	0.22 ***	0.04	0.12 *	−0.09	−0.10*	0.07	---	
9. T3 Compliance	−0.12 *	−0.04	0.69 ***	0.56 ***	0.13 **	−0.01	0.06	0.08	(0.83)

Values on the diagonal (in parentheses) are Cronbach’s alpha coefficients. * *p* < 0.05, ** *p* < 0.01, *** *p* < 0.001.

**Table 3:** Latent class models comparison.

Model	Class Size	Entropy	LMR LRT
2 Classes	68% 32%	0.97	1397.19 ***
3 Classes	64% 27% 09%	0.98	565.25 **
4 Classes	60% 21% 11% 08%	0.96	301.27
5 Classes	51% 16% 13% 13% 07%	0.98	239.45

LMR LRT = Lo–Mendell–Rubin Likelihood Ratio Test. *** *p* < 0.001, ** *p* < 0.01.

**Table 4:** Descriptive statistics for latent class membership.

	Class 1	Class 2	Class 3
Individuals in Class	114	258	37
Females	44%	40%	46%
Mean Age (SD)	39.01 (10.46)	39.61 (10.43)	36.76 (11.24)
Racial/Ethnic Minorities	30%	22%	49%
Mean Per Capita Income (SD)	3.08 (1.82)	3.45 (2.04)	1.94 (1.21)
College Graduates	45%	50%	40%
Mean Job Insecurity (SD)	2.52 (0.49)	0.25 (0.37)	1.23 (0.82)
Mean Financial Inadequacy (SD)	1.82 (0.64)	1.34 (0.42)	3.35 (0.61)
Mean Compliance with CDC Guidelines (SE)	3.81 (0.06)	3.95 (0.04)	3.73 (0.13)

To compute descriptive statistics presented in this table, participants were assigned to a latent class based on their highest posterior probability of class membership (i.e., assuming that classification error was null). Hence, besides the last row, which refers to the output of the BCH method (see hypothesis 1 in-text), this table should be considered exploratory and interpreted with caution.

**Table 5:** Parameter estimates for the model predicting compliance.

Predictors	Class 1		Class 2		Class 3	
	Estimate	SE	Estimate	SE	Estimate	SE
Attitudes	0.38 **	(0.13)	0.49 ***	(0.06)	0.19	(0.10)
Worry	0.21 *	(0.10)	0.09	(0.06)	0.49 ***	(0.14)
Health Conditions	0.01	(0.08)	−0.03	(0.05)	−0.04	(0.05)
COVID-19 Impact	0.00	(0.04)	0.05	(0.03)	−0.15	(0.10)
Per Capita Income	−0.02	(0.03)	−0.03	(0.02)	−0.06	(0.06)
Education	0.01	(0.04)	−0.01	(0.03)	0.07	(0.05)

* *p* < 0.05, ** *p* < 0.01, *** *p* < 0.001.

## Data Availability

The data presented in this study are available on request from the corresponding author. The data are not publicly available due to privacy concerns.

## Appendix A. Attitudes toward the CDC Guidelines

Please indicate your thoughts regarding physical/social distancing and hygiene recommendations from the CDC. (1. Strongly Disagree–7. Strongly Agree)

- It is important to maintain a distance of at least 6 ft. from others when out in public or at work.
- Staying at home except to conduct essential tasks (e.g., grocery shopping, medical appointments) is an effective way of stopping the spread of COVID-19.
- Disinfecting frequently used items and surfaces is beneficial to prevent spreading the virus.
- Frequently washing hands for a minimum of 20 s can reduce the spread of the virus.
- Avoiding touching my face will help protect me from COVID-19.
- When coughing or sneezing, people should aim inside their elbow or into a tissue.
- The economic cost of social distancing measures is worth the price to protect public health.
- Wearing a mask while out in public can reduce my chances of potentially spreading COVID-19 to others.

## Appendix B. Worry of COVID-19 Scale

Thinking about COVID-19, please indicate the extent to which you agree or disagree with the following statements. (1. Strongly Disagree–7. Strongly Agree)

- COVID-19 poses a serious threat to public health.
- I am worried about catching COVID-19.
- I am concerned about my family members or close others being exposed to the COVID-19 virus.
- I worry that I might unintentionally spread COVID-19 to others.
